# Stress, Sleep Quality, and Academic Performance among Dental Students in Shiraz, Iran

**DOI:** 10.1155/2022/3781324

**Published:** 2022-05-29

**Authors:** Zahra Jowkar, Zahra Fattah, Zahra Khorshidi Asl, Seyed Ahmadreza Hamidi

**Affiliations:** ^1^Oral and Dental Disease Research Center, Department of Operative Dentistry, School of Dentistry, Shiraz University of Medical Sciences, Shiraz, Iran; ^2^Department of Operative Dentistry, School of Dentistry, Shiraz University of Medical Sciences, Shiraz, Iran

## Abstract

**Background:**

Quality of sleep and stress level can affect the health, capacity of learning, and academic performance of the students. This study aimed to investigate the association between stress and sleep quality with academic performance among undergraduate clinical dental students in Shiraz, Iran.

**Methods:**

This cross-sectional study was conducted during the second semester of the academic year 2020–2021 among clinical dental students at Shiraz Dental School, Iran. A total of 138 students completed Pittsburgh Sleep Quality Index (PSQI) and dental environment stress (DES) questionnaire. The grade point averages (GPAs) of the previous terms of the participants were also collected. Data analysis was performed using Kolmogorov–Smirnov test, the one-way ANOVA, post hoc Duncan's test, nonparametric Kruskal–Wallis H test, Wilcoxon–Mann–Whitney test, and the chi-squared test. The *p* values of less than 0.05 were considered statistically significant.

**Results:**

Clinical dental students participated in this study experienced moderate levels of stress and poor sleep quality. Mean total DES and PSGI scores did not differ by sex, year of study, marital status, and place of residence (*p* values >0.05). Most of the students (52.9%) had moderate GPAs. A significant relationship was observed between sex and GPA as well as between place of residence and GPA (*p* values <0.05). No significant differences were found between DES total score or PSQI score and GPA categories (*p* values >0.05). A significant direct relationship between DES total score and PSQI score was observed (*p* < 0.05).

**Conclusion:**

Dental undergraduates in Shiraz, Iran, experienced moderate levels of stress and poor sleep quality. The results showed no significant difference between sleep quality or DES and academic achievement. However, a significant direct correlation was observed between sleep quality and dental environment stress.

## 1. Introduction

One of the most important pivotal factors in human health and life is sleep, which plays a direct role in learning, practice, and physical and mental health [1]. Insufficient sleep has adverse health and behavior consequences such as daytime sleepiness, feeling irritable during the day, and depressed mood [2, 3]. Reduced attention, psychomotor vigilance, and increased variability in behavioral responses are the most consistent effect of sleep deprivation [4]. Students may be at risk of developing sleep disorders during their education. Sleep disorders may negatively affect the academic performance of the students because inadequate sleep can adversely affect the neurobehavioral functions and learning capacity of the students [1, 5]. After previous academic achievement and class attendance, sufficient sleep was found as the third most important variable in predicting semester grades [6].

Dental schools are considered to be stressful learning environments [7]. Dental students need to acquire and apply a variety of proficiencies such as theoretical knowledge and clinical and interpersonal communication skills during their studies at university [8]. The students' well-being may be affected by persistent stress, which can impair the students' efficiency at work or their learning. Persistent stress of the students may also lead to difficulty to interact with patients, inability to continue working, depersonalization, and finally deteriorating the students' academic achievements [9]. Higher levels of stress were found for dental students in clinical years (fourth, fifth, and sixth) compared to dental students in preclinical years in a previous study [10].

No previous study assessed the relationship between sleep quality, dental environment stress, and academic performance among clinical dental students at Shiraz University of Medical Sciences. Thus, this study aimed to evaluate the relationship among sleep quality, dental environment stress, and academic performance, among clinical dental students at Shiraz University of Medical Sciences.

## 2. Methods

This was a cross-sectional study, which was performed during the second semester of the academic year 2020–2021, among the dental students of the fourth, fifth, and sixth years of education at Shiraz Dental School, Shiraz, Iran. The convenience sampling method was used to select the participants. The dentistry-specific studies begin in the third year of education at Shiraz Dental School and the year 4–year 6 students are in the clinical stage.

The study protocol was approved by the Medical Ethics Committee of Shiraz University of Medical Sciences (IR.SUMS.DENTAl.REC.1399.201), and all participants signed a written consent form before enrolling in the study. Two questionnaires were delivered to the students along with a cover letter containing the objectives of the study, voluntary participation, and the confidentiality of the data. The total number of the year 4–year 6 students (clinical dental students) at Shiraz Dental School was 200. A total of 138 dental students (69% of the total students who were in the clinical stage) participated in the present study. The needed sample size was calculated by a biostatistician. The sample size was determined based on the expectation of observing a correlation for *α* level of 0.05 and power of 0.80% and calculated by sample size formula. According to the mentioned parameters, a sample size of 46 for each year of study (4, 5, and 6) was determined as appropriate. Thus, the total number of the sample size was 138.

Dental Environment Stress (DES) questionnaire was used to assess stress among dental students [11]. A printed Persian version of the DES questionnaire validated by Ramazani and Nazari was used in this study [12]. A total of 32 items related to the stressors associated with undergraduate dental training are grouped under six main stress-provoking domains in this questionnaire. The main domains include academic efficiency (7 items), patient treatment (4 items), internal beliefs (4 items), academic factors (2 items), clinical education (11 items), and other items (4 items) [12]. A 4-point Likert scale (1 = not stressful, 2 = slightly stressful, 3 = moderately stressful, and 4 = very stressful) was used to rate each item of the questionnaire. Then, the mean score was calculated for each item. The mean score of less than 42 shows that the dental environment stress is mild. The mean score of 42 to 84 and more than 84 mean that the dental environment stress is moderate and high, respectively [12–14]. The demographic information of the participants (age, gender, the academic year in dentistry, marital status, and place of residence including the dormitory, with parents, and individually) were also recorded using a demographic form.

To evaluate the academic achievement, the grade point averages (GPAs) of the previous terms of the participants were collected in the demographic form. GPA is from 0 to 20 in the Iranian educational system. The academic achievement of the participants was classified as good (a GPA of 17 or higher), moderate (a GPA of 14–16.99), and poor (a GPS of less than 13.99) based on the recorded GPAs [15].

Pittsburgh Sleep Quality Index (PSQI), which is a self-report questionnaire, was used for the assessment of the quality of sleep [16]. PSQI contains 18 questions, which are classified into seven components. The first component (Question 9) is the subjective sleep quality. The second component (Question 2 and part of Question 5) assesses delays in falling asleep. The third component (Question 4) is related to sleep duration. The fourth component deals with the efficiency and effectiveness of sleep. Total hours of sleep is divided by total hours in the bed, and then, the obtained number is multiplied by 100 to calculate the score of the fourth component. The fifth (mean value of Question 5) and sixth components (Question 6) are related to sleep disorders and hypnotic drugs, respectively. The seventh component (mean scores of Questions 7 and 8) evaluate inadequate performance throughout the day. Each question is rated on a 3-point scale ranging from 0 (0 = “not at all”) to 3 (maximum score for each component). Therefore, the total score of the seven components can range from 0 to 21. Higher scores are related to lower sleep quality and a score above 5 is considered as poor sleep quality. The reliability and validity of the Persian form of PSQI have also been approved in Iran in a previous study [17].

SPSS software (version 16.0; SPSS, Chicago, IL) was used for data analysis. The normality of the data was checked based on the Kolmogorov–Smirnov test. Afterward, data analysis was performed using the one-way ANOVA, post hoc Duncan's test, nonparametric Kruskal–Wallis H test, Wilcoxon–Mann–Whitney test, and the chi-squared test. The *p* values of less than 0.05 were considered statistically significant.

## 3. Results

A total of 138 clinical dental students participated in this study. The mean age of the participants was 23.13 ± 1.58 years (ranging from 21 to 30 years old). The number of the participants from each year dental students (fourth-, fifth-, and sixth-year) was 46. 51.2% (*n* = 71) of participants were females, and 48.6% (*n* = 67) were male. The percent of the students who lived in dormitories, with their family, and individually were 31.2% (*n* = 43), 52.2% (*n* = 72), and 16.6% (*n* = 23), respectively.

The mean scores of the 6 main domains of the DES questionnaire are shown in Table 1. The normality assumption was held in all cases. The mean DES total score for clinical dental students was 71.08 ± 14.49, which exhibited that clinical dental students in Shiraz, Iran, experienced moderate levels of stress.

The mean DES total score for female and male students was 74.08 ± 14.00 and 67.91 ± 14.43. The chi-squared test revealed no significant differences between the mean DES total score of male students and that of the female students (*p*=0.23).

Moreover, the mean total scores of DES for fourth-, fifth-, and sixth-year dental students were 67.5, 74.65, and 71.06, respectively. The ANOVA showed that mean total DES scores did not differ by year of study (*p*=0.062). According to the ANOVA test, significant differences were found in the scores of “patient treatment” and “academic factors” among the students in different study years (*p*=0.04 and 0.007, respectively). The post hoc Duncan's test ranked the means in two different subsets at *p* < 0.05. Dental students of the fourth year of education demonstrated significantly lower stress scores for patient treatment and academic factors compared to those of the fifth- and sixth-year dental students (*p* < 0.05). However, no significant differences were observed among the students with different levels of education in terms of the stressors related to other main domains of the DES questionnaire (*p* values >0.05). DES total score did not differ by marital status and place of residence according to the chi-squared test (*p* values >0.05).

The mean scores of the 7 main components of the PSQI are presented in Table 2. The mean PSQI total score for clinical dental students was 5.76, which exhibited that clinical dental students in Shiraz, Iran, experienced poor sleep quality. ANOVA revealed no significant differences in the quality of sleep among the students with different academic levels (*p*=0.91). The nonparametric Kruskal–Wallis H test was used to analyze the differences among the 7 components of PSQI based on the year of education. It revealed that there was a significant difference among the students with different academic levels in terms of sleep duration (*p*=0.22). However, no significant differences were observed among the students with different levels of education in terms of other components of PSQI (*p* values >0.05). Dunn's post hoc test showed that the fourth-year dental students had the highest sleep duration and the sixth-year dental students reported the lowest sleep duration (*p* values< 0.05).

The mean PSQI total scores for single and married dental students were 5.88 ± 23 and 4.64 ± 73. 46.8% of single students and 21.4% of married students experienced poor sleep quality. Wilcoxon–Mann–Whitney test showed no statistically significant differences between the mean PSQI total scores of the single and married students (*p*=0.08). No significant relationships were observed among the 7 components of PSQI based on the marital status of the participants (*p* values >0/05).

The mean PSQI total scores for male and female dental students were 5.55 ± 0.33 and 5.95 ± 0.29, respectively. No statistically significant difference was observed between the mean PSQI total scores of the male and female students (*p*=0.18). According to the Wilcoxon–Mann–Whitney test, male students had significantly lower sleep duration compared to the female one (*p*=0.04). No significant relationships were observed among other components of PSQI based on the sex of the participants (*p* values >0/05).

Wilcoxon–Mann–Whitney test showed no statistically significant differences between the mean PSQI total scores of the students based on the place of residence of the students (*p*=0.76).

The grade point averages (GPAs) of the previous terms of the participants were collected and used for assessing academic achievement. GPA scores of the participants based on year of study, gender, marital status, and place of residence are presented in Table 3. Most of the students (52.9%) had moderate GPAs. 33.3% of the participants had good GPAs. Poor GPAs were observed in 13.8% of the students. The chi-squared test demonstrated a significant relationship between sex and GPA (*p*=0.003). More female students had good GPAs compared to male students. However, more male students had moderate and poor GPAs compared to female ones.

The chi-squared test showed no significant differences between the year of study or marital status and GPA category (*p* values >0.05). According to the chi-squared test, there was a significant relationship between place of residence and GPA categories (*p*=0.03). More dental students who lived with their families demonstrated good GPAs compared to the students who lived in dormitories or individually. Most of the students who lived in dormitories or individually have moderate or poor GPAs.

No significant differences were found between DES total score or PSQI score and GPA categories according to the chi-squared test (*p* values = 0.22 and 0.44, respectively).

The chi-squared test showed that there was a significant direct relationship between DES total score and PSQI score (*p*=0002). More students who experienced poor sleep quality demonstrated high levels of dental environment stress compared to the students who did not experience poor sleep quality. Additionally, most of the students with moderate levels of dental environment stress (61.9%) did not have sleep disorders. However, most of the students with high levels of dental environment stress (72.0%) experienced good sleep quality.

## 4. Discussion

The present study was conducted to explore whether sleep quality and dental environment stress can affect academic performance or not among clinical dental students at Shiraz University of Medical Sciences. According to the results of this study, clinical dental students in Shiraz, Iran, experienced moderate levels of stress and poor sleep quality. Moreover, a significant direct relationship between the DES total score and PSQI score was found.

Pittsburgh Sleep Quality Index (PSQI) was used for the assessment of the quality of sleep in this study. According to the results of this study, clinical dental students in Shiraz, Iran, experienced poor sleep quality.

Generally, it has been reported that sleep and learning are two poles of dynamic interaction, which can mutually affect each other [18]. A systematic review study reported that sleep disturbance adversely affected academic performance, general health, and social status of the students [3]. Although there is a possibility of sleep disorders in dental students, there is no certainty about the relationship between poor sleep quality and the lack of academic achievement [1, 19, 20]. Some studies reported an inverse relationship between poor sleep quality and academic performance [20–22]. However, the present study did not identify a significant relationship between sleep quality and academic performance of Shiraz clinical dental students. In line with findings of the present study, no causal relationship was found between sleep quality and academic performance in a previous systematic review [18]. Therefore, the findings of different studies regarding the relationship between academic performance and sleep quality of the students are not the same. The reason for this difference is the existence of uncontrollable variables, which may affect academic success in different studies. Some of these factors include social issues, family size, the evolutionary process, social media dependency, intake of supplements and vitamins, level of family income, and addiction to social networks [1].

Another finding of the present study was that fourth-year dental students had the highest sleep duration and fifth- and sixth-year dental students reported the lowest sleep duration. This finding may be related to the higher number of clinical courses offered to fifth-year and sixth-year dental students compared to those offered to the fourth-year dental students at Shiraz Dental School. The results of the present study also showed that male students had significantly lower sleep duration compared to female ones. This finding was in line with that of a previous study, which reported lower average sleep duration and more sleep problems for males compared to females [23]. However, year of study, sex, marital status, and place of residence did not affect PSQI in the present study.

Another variable, which was assessed in the present study, was dental environment stress. Dental school is a highly stressful environment, and dental students demonstrated higher levels of stress than medical students [24]. A previous study on first-year dental students showed that high stress levels in students were correlated with lower ratings of physical and emotional health and lower GPAs compared to the students with lower stress levels [13]. Additionally, high levels of stress in students may lead to greater medical errors, lower quality of patient's care, and a decrease in patient satisfaction [25]. The mean DES total score for clinical dental students was 71.08 in the present study, which exhibited that clinical dental students in Shiraz, Iran, experienced moderate levels of stress. This finding was in line with the results of a previous study, which was conducted in Fujian, China, and reported that the stress experienced by dental undergraduate students was moderate [19]. This finding can be justified by the fact that dental students face various challenges during their education such as dealing with patients and learning both the theoretical and technical aspects of the dental curriculum [7].

Different correlations were previously reported by various studies regarding the year of study and stress level among dental students. Longitudinal stress of dental students was assessed in a previous study. It was observed that the stress levels of the students increase in the last years of education compared to the first year [26]. Conversely, the third-year students demonstrated the highest amount of stress compared to the other students in a previous study [27]. Another study reported the highest and lowest levels of stress among dental students in final-year students and first-year students, respectively [13]. This finding was in contrast to the findings of the present study, which showed no differences among stress levels of dental students regarding the year of education.

Differences in stress levels between men and women were found in some previous research, while others did not report significant differences between the stress level of female students and that of male students [7, 28–30]. No significant differences were found between the stress levels of female and male students in the current study. The difference among the results of the various studies can be explained by differences in the studied populations and various sociocultural factors.

While marriage and raising children during academic education years may lead to a high level of stress among the students, being single has also been reported as a stress factor previously [29, 31]. The differences in the spiritual support that married students receive from their families, the differences in family conditions, and the differences in the sample sizes of the married and single students in different studies can be attributed to the inconsistency between the results of different studies [7, 29, 31]. No significant differences were found among stress levels of married and single students in the current study. However, the number of married students was much lower than that of the single ones in the current study, and thus, it was not possible to accurately interpret the relationship between students' marital status and their level of stress.

Another variable that was assessed in the present study was academic performance of Shiraz dental clinical students with recording their GPAs. Several factors may affect academic achievement of the students such as diagnosed depression and/or anxiety, learning disabilities, and study habits [15, 32]. The results of the present study revealed that the GPAs of most of the students were between 14 and 16.99. Only 13.8% of the students had poor GPAs. Year of study or marital status did not influence the GPA category. More female students had good GPAs compared to male students in the present study. In line with the results of the present study, a previous study reported higher GPAs for female students compared to those of male students [14].

Another finding of the current study was that more dental students who lived with their family demonstrated good GPAs compared to the students who lived in dormitories or individually. Most of the students who lived in dormitories or individually have moderate or poor GPAs. Better GPAs of the students living with their families compared to the other ones may be attributed to the support that the students receive from their families.

The present study also assessed the relationship between sleep quality, dental environment stress, and academic performance, among Shiraz clinical dental students. No significant differences were found between DES total score or PSQI score and GPA categories. A previous study conducted in Fujian, China, reported that the level of stress was negatively correlated with GPA score [14]. However, no significant differences were found between DES total score and GPA categories in the present study. This difference may be related to the differences in the studied population and their socioeconomic and cultural differences.

This study revealed a significant direct relationship between DES total score and PSQI score. More students who experienced poor sleep quality demonstrated high levels of dental environment stress compared to the students who did not experience poor sleep quality. Most of the students with moderate levels of dental environment stress did not have sleep disorders. However, most of the students with high levels of dental environment stress experienced poor sleep quality. This finding was in line with findings of a previous study, which showed a significant association between stress and sleep quality among medical students [33]. Inadequate sleep may be correlated with poor perceived mental health and depression or depressive symptoms [3]. On the other hand, stress may result in many sleep difficulties, such as midsleep awakening, waking up too early, and restless sleep [34]. Dental students may reduce their sleeping time to have extra hours for studying, and therefore, they may not consider sleep as a top priority during their education.

According to the results of this study, clinical dental students in Shiraz dental school experienced moderate levels of stress and poor sleep quality. Moreover, a direct relationship was found between poor sleep quality and dental environment stress in this study. It is necessary to design additional research to explore potential causes for poor sleep quality. Moreover, it will be helpful to educate dental students about sleep hygiene and the negative consequences of poor sleep quality. An organized stress reduction program should also be instituted at the Shiraz Dental School.

This study has some limitations. In the present study, data were gathered via questionnaires, which relied on self-report data. Moreover, as with other studies calculating the impact of various health factors on specific outcomes, the findings of the present study may suffer from the multicollinearity problem [32]. Additionally, to find a causal relationship between stress level in the dental environment and sleep quality and the academic achievement of the students, prospective, longitudinal studies should be designed.

## 5. Conclusion

Dental undergraduates in Shiraz, Iran, experienced moderate levels of stress and poor sleep quality. The results showed no significant difference between sleep quality or DES and academic achievement. However, a significant direct correlation was observed between sleep quality and dental environment stress.

## Figures and Tables

**Table 1:** The mean scores of the 6 domains of the DES questionnaire.

Domains	Year of study (number)	Scores (mean ± SD)
I-Academic efficiency	4 (46)	15.50 ± 2.93
	5 (46)	16.58 ± 3.31
	6 (46)	15.10 ± 3.92
	Total (138)	15.73 ± 3.44

II-Patient treatment	4 (46)	8.47 ± 2.21
	5 (46)	9.60 ± 2.44
	6 (46)	9.60 ± 2.83
	Total (138)	9.23 ± 2.54

III-Internal beliefs	4 (46)	7.89 ± 2.70
	5 (46)	8.69 ± 3.33
	6 (46)	8.23 ± 3.50
	Total (138)	8.27 ± 3.19

IV-Academic factors	4 (46)	4.19 ± 1.61
	5 (46)	5.30 ± 1.90
	6 (46)	5.02 ± 1.65
	Total (138)	4.84 ± 1.78

V-Clinical education	4 (46)	22.95 ± 5.40
	5 (46)	25.89 ± 6.59
	6 (46)	25.15 ± 6.72
	Total (138)	24.66 ± 6.34

VI-Other items	4 (46)	8.52 ± 2.94
	5 (46)	8.56 ± 2.50
	6 (46)	7.93 ± 2.56
	Total (138)	8.34 ± 2.67

Total	4 (46)	67.54 ± 13.04
	5 (46)	74.65 ± 14.67
	6 (46)	71.06 ± 15.11
	Total (138)	71.08 ± 14.49

**Table 2:** The mean scores of the 7 components of the PSQI.

Components	Year of study (number)	Scores (mean ± SD)
1-Subjective sleep quality	4 (46)	1.04 ± 0.66
	5 (46)	1.13 ± 0.71
	6 (46)	0.95 ± 0.63
	Total (138)	1.04 ± 0.66

2-Delays in falling asleep	4 (46)	1.15 ± 0.91
	5 (46)	1.30 ± 0.98
	6 (46)	1.34 ± 0.92
	Total (138)	1.26 ± 0.28

3-Sleep duration	4 (46)	1.17 ± 0.82
	5 (46)	0.91 ± 0.72
	6 (46)	0.71 ± 0.71
	Total (138)	2.29 ± 0.75

4-Efficiency and effectiveness of sleeping	4 (46)	0.087 ± 0.28
	5 (46)	0.26 ± 0.71
	6 (46)	0.23 ± 0.56
	Total (138)	0.19 ± 0.51

5-Sleep disorders	4 (46)	0.98 ± 0.49
	5 (46)	0.98 ± 0.49
	6 (46)	0.98 ± 0.53
	Total (138)	0.98 ± 0.50

6-Hypnotic drugs	4 (46)	0.26 ± 0.77
	5 (46)	0.30 ± 0.75
	6 (46)	0.34 ± 0.73
	Total (138)	0.30 ± 0.75

7-Inadequate performance throughout the day	4 (46)	0.95 ± 0.81
	5 (46)	1.00 ± 0.86
	6 (46)	1.15 ± 0.89
	Total (138)	1.03 ± 0.85

Total	4 (46)	5.65 ± 2.48
	5 (46)	5.89 ± 3.00
	6 (46)	5.73 ± 2.32
	Total (138)	5.76 ± 2.6

**Table 3:** GPA scores of the participants based on year of study, gender, marital status, and place of residence.

Variable		GPA category/percent (number)		
		Good	Moderate	Poor
Year of study (number)	4 (46)	34.8% (16)	52.2% (24)	13.0% (6)
	5 (46)	30.4% (14)	52.2% (24)	17.4% (8)
	6 (46)	34.8% (16)	54.3% (25)	10.9% (5)

Gender	Female	45.1% (32)	47.9% (34)	7.0% (5)
	Male	20.9% (14)	58.2% (39)	20.9% (14)

Marital status	Single	33.1% (41)	53.2% (66)	13.7% (17)
	Married	35.7% (5)	50.7% (7)	14.3% (2)

Place of residence	Living in dormitories	23.3% (10)	55.8% (24)	20.9% (9)
	Living with the family	44.4% (32)	47.2% (34)	8.3% (6)
	Living individually	17.4% (4)	65.2% (15)	17.4% (4)

## Data Availability

The data that support the findings of this study are available on request from the corresponding author.
